# Using a Structured Environmental Assessment Tool of Inform Change Under Constraints: Action Marguerite

**DOI:** 10.1093/geroni/igaf122.623

**Published:** 2025-12-31

**Authors:** Robert Wrublowsky, Migette Kaup

**Affiliations:** RAW Consulting, Winnipeg, Manitoba, Canada; Kansas State University, Manhattan, Kansas, United States

## Abstract

One goal of the EASE is to assist care providers who have older, traditional buildings determine how to best utilize scarce resources when considering renovations that will have the most positive impact on residents, families and staff. Analysis of differences between settings that clearly reflect traditional design elements (long, double loaded corridors and minimal space for meaningful social engagement) and settings that have adopted some person-centered care values identify smaller scale and the presence of a functional kitchen as key factors. Both of these involve significant operational as well as environmental changes and can be met with resistance. This session describes the process of working with a care provider that had seven highly traditional living areas assessed with the EASE which resulted in identification of specific recommendations to reduce the size of living areas and include functional kitchens. The evidence-based nature of the EASE supports constructive responses to perceived barriers to adoption of changes that require operational changes. The EASE also identifies numerous low-cost interventions that can be easily adopted allowing for a phased implementation process.

